# Contrasting the Chromosomal Organization of Repetitive DNAs in Two Gryllidae Crickets with Highly Divergent Karyotypes

**DOI:** 10.1371/journal.pone.0143540

**Published:** 2015-12-02

**Authors:** Octavio M. Palacios-Gimenez, Carlos Roberto Carvalho, Fernanda Aparecida Ferrari Soares, Diogo C. Cabral-de-Mello

**Affiliations:** 1 UNESP—Univ. Estadual Paulista, Instituto de Biociências/IB, Departamento de Biologia, Rio Claro, SP, Brazil; 2 UFV–Univ. Federal de Viçosa, Centro de Ciências Biológicas, Departamento de Biologia Geral, Viçosa, MG, Brazil; Virginia Tech, UNITED STATES

## Abstract

A large percentage of eukaryotic genomes consist of repetitive DNA that plays an important role in the organization, size and evolution. In the case of crickets, chromosomal variability has been found using classical cytogenetics, but almost no information concerning the organization of their repetitive DNAs is available. To better understand the chromosomal organization and diversification of repetitive DNAs in crickets, we studied the chromosomes of two Gryllidae species with highly divergent karyotypes, i.e., 2n(♂) = 29,X0 (*Gryllus assimilis*) and 2n = 9, neo-X_1_X_2_Y (*Eneoptera surinamensis*). The analyses were performed using classical cytogenetic techniques, repetitive DNA mapping and genome-size estimation. Conserved characteristics were observed, such as the occurrence of a small number of clusters of rDNAs and U snDNAs, in contrast to the multiple clusters/dispersal of the H3 histone genes. The positions of U2 snDNA and 18S rDNA are also conserved, being intermingled within the largest autosome. The distribution and base-pair composition of the heterochromatin and repetitive DNA pools of these organisms differed, suggesting reorganization. Although the microsatellite arrays had a similar distribution pattern, being dispersed along entire chromosomes, as has been observed in some grasshopper species, a band-like pattern was also observed in the *E*. *surinamensis* chromosomes, putatively due to their amplification and clustering. In addition to these differences, the genome of *E*. *surinamensis* is approximately 2.5 times larger than that of *G*. *assimilis*, which we hypothesize is due to the amplification of repetitive DNAs. Finally, we discuss the possible involvement of repetitive DNAs in the differentiation of the neo-sex chromosomes of *E*. *surinamensis*, as has been reported in other eukaryotic groups. This study provided an opportunity to explore the evolutionary dynamics of repetitive DNAs in two non-model species and will contribute to the understanding of chromosomal evolution in a group about which little chromosomal and genomic information is known.

## Introduction

The Orthoptera order comprises more than 25,700 species, which form the most diverse group of polyneopteran insects [1,2,3]. Variability in the dipoid number has been documented in distinct lineages, which range in the most highly studied groups from 2n(♂) = 15 to 2n(♂) = 35 in katydids Tettigoniidae [4,5,6], from 2n(♂) = 8 to 2n(♂) = 23 in Acrididae grasshoppers [7,8,9] and from 2n(♂) = 7 to 2n(♂) = 29 in Gryllidae crickets [9,10,11,12,13], although the modal number of chromosomes has been documented, such as 2n(♂) = 23 in grasshoppers and 2n = 21 in Gryllidae crickets [8,9]. This extreme karyotypic divergence has been attributed to multiple major chromosomal restructuring events, such as centric fusions, tandem fusions, reciprocal translocations, dissociations and inversions involving autosomes and sex chromosomes [8,9]. In addition to these rearrangements being responsible for the variations in the diploid number, they also caused diversification of the sex chromosomes, originating distinct sex systems, such as neo-XY, neo-X_1_X_2_Y and X_1_X_2_0, with the origin attributed from the X0, which is considered atavic and modal for the group [8,9,14,15,16,17].

Large portions of eukaryotic genomes are composed of sequences that are repeated hundred to thousand times, which are called repetitive DNAs. These sequences could be tandemly arrayed as microsatellites, minisatellites and satellite DNAs (satDNAs) or occur in a scattered pattern, as transposons and retrotransposons [18,19,20,21,22]. In accordance with the selfish DNA hypothesis [23,24], some of these sequences have been maintained in the genome due to their ability to colonize genomic regions lacking or with a low level of recombination activity. Thus, these repeats tend to be highly abundant and ubiquitous in certain chromosomal compartments, such as telomeric and centromeric heterochromatin and non-recombining regions of the sex chromosomes. Repetitive DNAs generally are subjected to replication and expansion processes through multiple mechanisms, such as unequal cross-over, gene conversion, rolling-circle replication, whole-genome duplication, segmental duplications and transposition [18,21,25,26,27,28,29,30]. Because the size and abundance of the repeats vary greatly within and between species, repetitive DNAs are the major cause of variation in the size of eukaryote genomes, and they have also been involved in genomic evolution [18,31,32,33].

Orthopteran species frequently have large genomes, ranging in size from 1.52 Gb in the cave cricket *Hadenoecus subterraneus* [34] to 16.56 Gb in the grasshopper *Podisma pedestris* [35]. In some Orthopteran species, the genomes are rich in repetitive DNAs, as for example, in *Locusta migratoria* (genome size 6.3 Gb [36]) and *Schistocerca gregaria* grasshoppers (genome size 8.55 Gb [37]). Although the sizes of the genomes of cricket species are generally smaller than those of grasshoppers, they have large genomes compared with those of other insects (see [38,39]); however, very little information concerning the chromosomal organization of the repetitive DNAs in this group is available (see for example [13,17]).

Considering the large genomes rich in repetitive DNAs of some Orthopteran species and the possible role of these sequences in chromosomal evolution, in this study, we aimed to contribute to the understanding of the organization of repetitive DNAs in autosomes and their contribution to the structure of the derived sex chromosomes in crickets. For this purpose, we used two species belonging to the family Gryllidae that have highly divergent karyotypes, i.e., *Gryllus assimilis*, with 2n(♂) = 29,X0 [40], and *Eneoptera surinamensis*, with 2n(♂) = 9,neo-X_1_X_2_Y [12]. These genomes were compared using flow cytometric genome-size estimation, classical cytogenetic analysis and fluorescent *in situ* hybridization (FISH) using various repetitive DNAs as probes, such as multigene families (18S and 5S rDNA, U1 and U2 snDNAs and H3 histone), highly and moderately repetitive DNA fractions (*C*_*0*_ *t*-DNA), the classical insect telomeric repeat (TTAGG) and 16 microsatellite motifs.

## Materials and Methods

### Samples, classical chromosomal analysis and banding

Five male and seven female *Eneoptera surinamensis* were collected in the Parque Estadual Edmundo Navarro de Andrade (Rio Claro, SP, Brazil) between May 2013 and March 2014 with the authorization of COTEC (process number 341/2013) and were maintained in captivity until ovipositioning occurred, whereas *Gryllus assimilis* animals were obtained from a pool of individuals that had been bred in the biotery of the Univ. Estadual Paulista—UNESP (Rio Claro, SP, Brazil). The embryo preparations for chromosome obtaining were prepared according to Webb et al. [41], with slight modifications of the fixation materials, as follows: After hypotonization was accomplished, the same volume of Carnoy’s modified solution (3:1, absolute ethanol:acetic acid) was applied for 15 minutes, then the embryos were transferred to fresh Carnoy’s solution and were stored in a -20°C freezer until use. At least 60 embryos of each species were used for cytological preparations. In addition, four adult male testes of each species were dissected and were fixed in Carnoy’s modified solution. Adult specimens (ten of each species) were stored in 100% ethanol for subsequent DNA extraction.

Karyological studies were performed using conventional staining with 5% Giemsa solution to confirm the previous karyotypic descriptions. The C-banding procedure was conducted according to Sumner [42], and fluorochrome staining (CMA_3_/DA/DAPI) to identify G+C or A+T rich regions was performed as described by Schweizer et al. [43]. Female and male genomic DNA was extracted from femurs using the phenol/chloroform-based procedure described in Sambrook and Russel [44].

### Probes for repetitive DNAs

The DNA probes for the 5S rRNA and H3 genes were obtained through polymerase chain reaction (PCR) amplification using genomic DNA from *Abracris flavolineata* as a template and the primers described by Cabral-de-Mello et al. [45] and Colgan et al. [46] for the 5S rRNA gene and the H3 gene, respectively. The sequences of the U snDNAs of *Rhammatocerus brasiliensis* were obtained using the primers described by Cabral-de-Mello et al. [47] for U1 snDNA and those of Bueno et al. [48] for U2 snDNA. These sequences were previously used as probes and are deposited in GenBank under accession numbers KC936996 (5S rDNA), KC896792 (H3 histone gene), KC896793 (U1 snDNA) and KC896794 (U2 snDNA). The 18S rDNA probe was obtained from a cloned fragment previously isolated from the genome of the *Dichotomius semisquamosus* beetle (GenBank accession number GQ443313 [45]).

The repetitive DNA-enriched samples were obtained based on the renaturation kinetics of *C*_*0*_ *t*-DNA (DNA enriched for highly and moderately repetitive DNA sequences), according to the protocol of Zwick et al. [49], with modifications [45]. It was denaturated 150 ng/μL of fragmented genomic DNA at 95°C. The reassociation time of 25 min was used for both species. The DNA was purified/extracted using a traditional phenol-chloroform procedure [44].

The telomeric probe was obtained through PCR using the self-complementary primers (TTAGG)_5_ and (CCTAA)_5_ according to Ijdo et al. [50]. Finally, the probes for the microsatellite motifs were directly labeled at the 5’ end with biotin-14 dATP (Sigma-Aldrich, St Louis, MO, USA) during their synthesis, including probes for mononucleotides (A)_30_ and (C)_30_, dinucleotides, (CA)_15_, (CG)_15_, (TA)_15_ and (AG)_10_, trinucleotides (CAA)_10_, (CAC)_10_, (TAA)_10_, (GAA)_10_, (CGG)_10_, (CAG)_10_, (TAC)_10_ and (GAG)_10_, tetranucleotides (GACA)_4_ and (GATA)_8_.

### Fluorescence *in situ* hybridization (FISH)

The non-cloned 5S rDNA and U snDNAs sequences and telomeric probes were labeled through PCR using digoxigenin-11-dUTP (Roche, Mannheim, Germany). Plasmids containing the 18S rRNA gene or H3 histone gene and the *C*_*0*_ *t*-DNA fraction were labeled via nick translation using biotin-14-dATP (Invitrogen, San Diego, CA, USA). Single or two-color FISH of mitotic cells was performed according to Pinkel et al. [51], with modifications [45]. Fiber-FISH was conducted as described in de Barros et al. [52] and Camacho et al. [53] using suspensions of testis cells. Probes labeled with digoxigenin-11-dUTP were detected using rhodamine-conjugated anti-digoxigenin (Roche), and probes labeled with biotin-14-dATP were detected using Streptavidin Alexa Fluor 488-conjugated (Invitrogen).

The preparations were counterstained using 4’,6-diamidine-2’-phenylindole dihydrochloride (DAPI) and were mounted in Vectashield (Vector, Burlingame, CA, USA). The chromosomes and signals were observed using an Olympus microscope BX61 equipped with a fluorescence lamp and the appropriate filters. Grey-scale images were captured using a DP70 cooled digital camera and were processed using Adobe Photoshop CS2 software.

### Genome size estimation

The flow cytometric (FCM) analyses were conducted as described by Lopes et al. [54], in the Laboratory of Cytogenetics and Cytometry, Department of General Biology, Universidade Federal de Viçosa (UFV). The nuclear DNA contents of adult *G*. *assimilis* and *E*. *surinamensis* males and females were determined using the C DNA content of a *Scaptotrigona xantotricha* female as an internal standard, which was confirmed by comparison with that of the international standard, *Drosophila melanogaster*.

To prepare the nuclear suspensions for FCM, brain ganglia were excised from the standard animal and the sample animals in physiological saline solution (0.155 mM NaCl). The samples consisted of three males and three females of each species, and each individual was manipulated and analyzed separately, representing three independent repetitions. The materials were simultaneously crushed 10–12 times in a tissue grinder using a pestle (Kontes Glass Company, NJ, USA) in 100 μL of OTTO-I lysis buffer [55] containing 0.1 M citric acid (Merck, NJ, USA), 0.5% Tween 20 (Merck) and 50 μg mL-1 RNase A (Sigma-Aldrich), pH = 2.3. The suspensions were adjusted to 1.0 mL using the same buffer, filtered through a 30-μm nylon mesh (Partec, Nuremberg, Germany) and centrifuged at 100 g in microcentrifuge tubes for 5 min. The pellets were then incubated for 10–15 min in 100 μL of OTTO-I lysis buffer and were stained using 1.5 mL of OTTO-I:OTTO-II (1:2) solution [56,57] supplemented with 75 μM propidium iodide (PI) (excitation/emission wavelengths: 480-575/550-740 nm, [58]) and 50 μg mL-1 RNase A (Sigma-Aldrich), pH = 7.8. The nuclear suspensions were filtered through a 20-μm nylon mesh filter (Partec) and were maintained in the dark for 30 min.

The suspensions were analyzed using a Partec PAS flow cytometer (Partec) equipped with a laser source (488 nm). PI fluorescence emitted by the nuclei was collected using an RG 610-nm band-pass filter and converted to 1024 channels. The equipment was calibrated for linearity and was aligned using microbeads and standard solutions according to the manufacturer’s recommendations. FlowMax software (Partec) was used for data analysis. The standard nuclear peak was set to channel 100 and more than 10,000 nuclei were analyzed. Three independent replications were conducted, and histograms with a coefficient of variation (CV) of greater than 5% were rejected.

## Results

**Fig 1:**
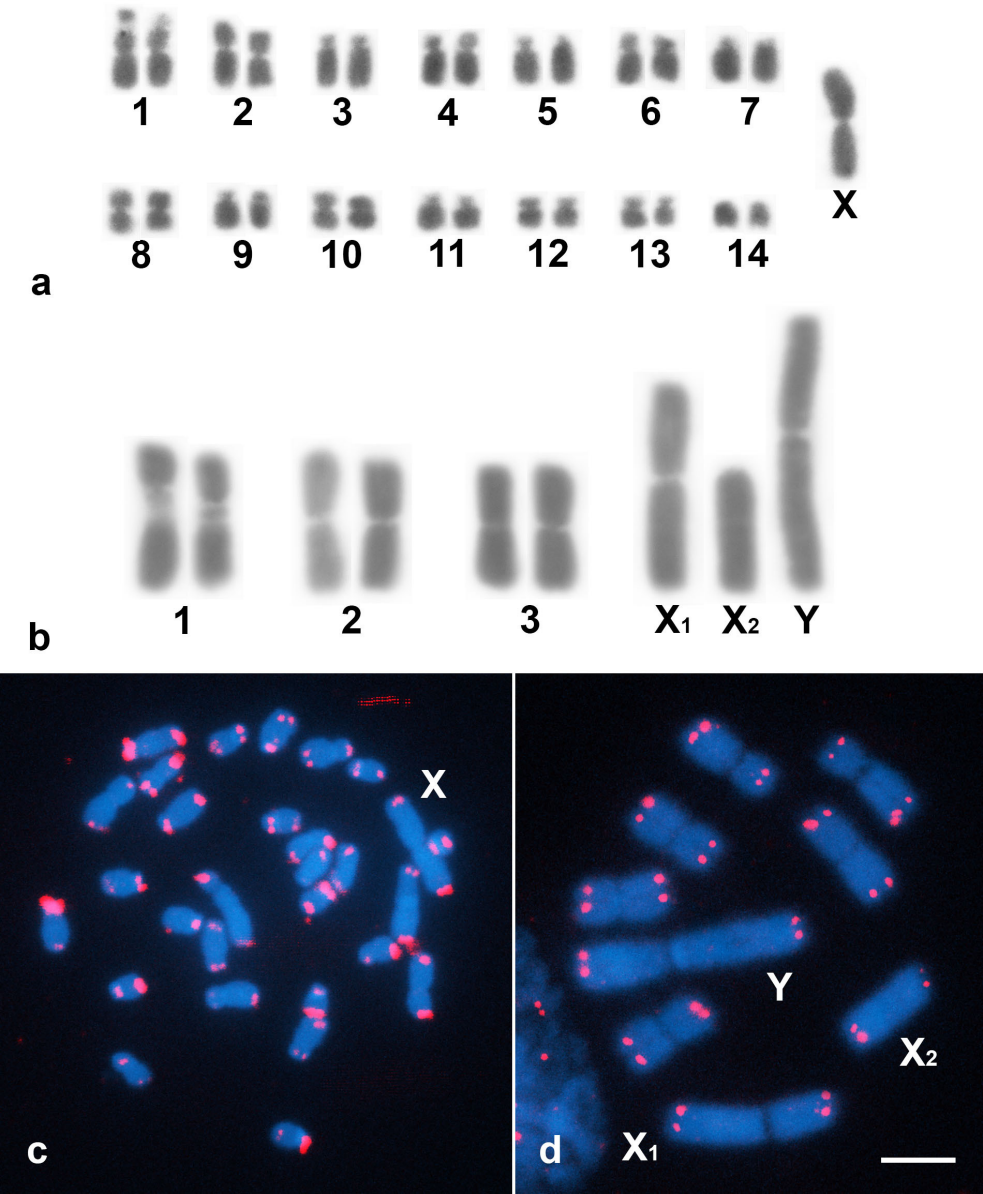
Male karyotypes of *Gryllus assimilis* (a) and *Eneoptera surinamensis* (b) observed using Giemsa staining. The chromosomes of both species were arranged in descending order of size. FISH analysis of the telomeric repeat in the mitotic cells of *G*. *assimilis* (c) and *E*. *surinamensis* (d). The sex chromosomes are indicated in the figure. Bar = 5 μm.

The karyotype of *Gryllus assimilis* is 2n = 29♂/30♀, with a X0♂/XX♀ sex-chromosome system (Fig 1A). The autosomes consist of four pairs of metacentric (1, 2, 8 and 10) chromosomes, four pairs of submetacentric (4, 6, 12 and 13) chromosomes, five pairs of subtelocentric (3, 5, 7, 9 and 11) chromosomes and one pair of telocentric (14) chromosomes. The X chromosome is metacentric and is the largest chromosome of the karyotype. One evident interstitial secondary constriction was observed in the metacentric pair of chromosome 1 (Fig 1A). Analysis of *E*. *surinamensis* males and females revealed a karyotype of 2n = 9♂/10♀ with a neo-X_1_X_2_Y♂/X_1_X_1_X_2_X_2_♀ sex-chromosome system. Autosomal pairs 2 and 3 are metacentric chromosomes, whereas pair 1 are submetacentric chromosomes with a conspicuous proximal secondary constriction localized on the long arm. The neo-X_1_ chromosome is metacentric, the neo-X_2_ chromosome is telocentric and the neo-Y chromosome, which is submetacentric, is the largest sex chromosome (Fig 1B). These results are similar to the previously described results for both species [12,40]. In both species, only terminal sites of the autosomes and sex chromosomes were recognized using the telomeric probe (Fig 1C and 1D).

**Fig 2:**
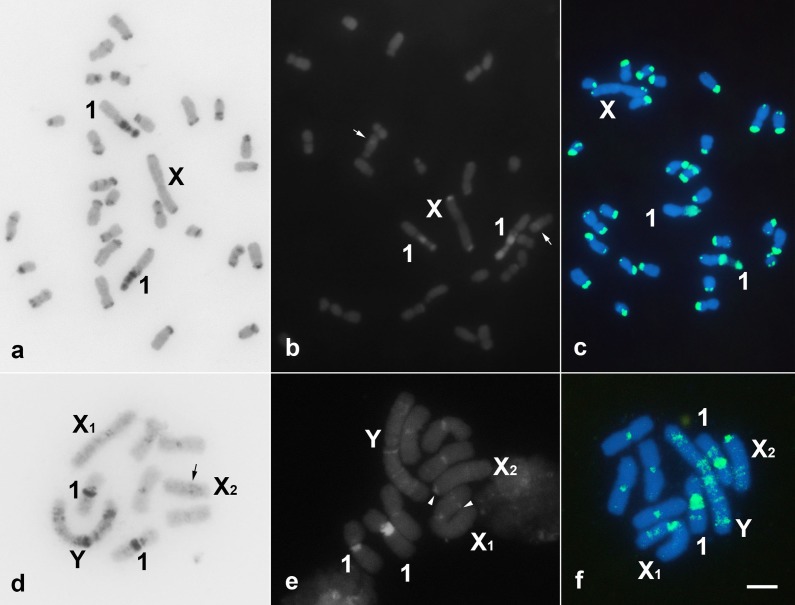
C-banding (a, d), CMA_3_ fluorochrome staining (b, e) and FISH of the *C*_*0*_ *t*-DNA (c, f) on mitotic chromosomes of *G*. *assimilis* (a, b, c) and *E*. *surinamensis* (d, e, f) cells. The sex chromosomes and the chromosomes bearing the secondary constrictions (pair 1) are indicated in each of the panels. Note the predominance of terminal C-positive blocks in *G*. *assimilis* and *E*. *surinamensis* chromosomes, the neo-Y chromosome with some heterochromatic blocks and CMA_3_^+^ signals along its entire length, as well as the *C*_*0*_ *t*-DNA signals. In (b), the white arrow indicates the interstitial CMA_3_^+^ block, and in (d), the black arrow indicates the interstitial C-positive block in the neo-X_2_ chromosome. In (e) arrowheads indicate the CMA_3_^+^ signals in C-negative regions of the neo-X_1_ and neo-X_2_. One chromosome is missing in (b). Bar = 5 μm.

The C-positive regions were observed in distinct areas for the distinct chromosomes of *G*. *assimilis*, with more frequent terminal blocks on the short chromosomal arms and in the pericentromeric regions. The X chromosome harbored only C-positive terminal blocks in both arms. The secondary constriction of chromosomes 1 was intensely stained via C-banding (Fig 2A). In *E*. *surinamensis*, all of the chromosomes except the neo-X_2_ chromosome exhibited pericentromeric C-positive bands. The neo-X_2_ chromosome had a faint interstitial C-positive band, and the pair 1 chromosomes exhibited a heterochromatic block coincident with the secondary constriction. Additionally, some dispersed C-positive blocks were observed in the neo-Y chromosomes (Fig 2D). Most of the C-positive blocks in the *G*. *assimilis* chromosomes were neutral for G+C or A+T base pairs. A remarkable number of G+C positive blocks (CMA_3_^+^) were observed only in the secondary constriction of the chromosome pair 1 and in the terminal region of the X chromosome, although faint CMA_3_^+^ signals were also observed in a few other chromosomes, in terminal or interstitial positions (Fig 2B). In the *E*. *surinamensis* chromosomes, all of the C-positive blocks were CMA_3_^+^ and the neo-X_1_ and neo-X_2_ chromosomes exhibited C-negative blocks rich in G+C base pairs, which were interstitial in the short arm and in the pericentromeric region, respectively (Fig 2E). No DAPI positive blocks were observed (result not shown). In both species, C-positive regions were also identified in the *C*_*0*_ *t*-DNA fractions (Fig 2C and 2F). Additionally, in the *E*. *surinamensis* cells, faint signals were observed distributed along some of the chromosomes in the C-negative regions, which include the pericentromeric area of the neo-X_2_ chromosome (Fig 2F).

**Fig 3:**
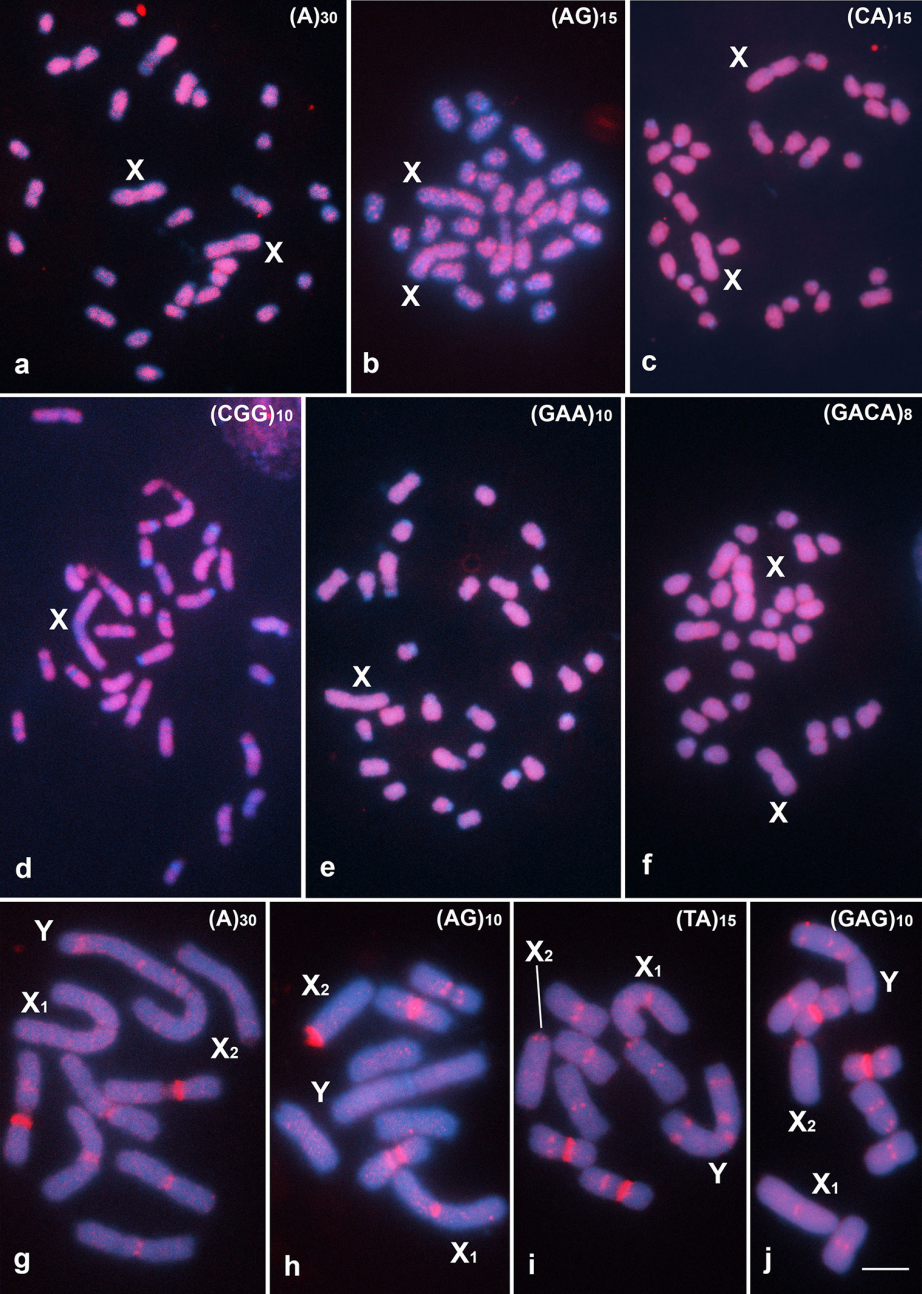
Mitotic metaphase cells of *G*. *assimilis* (a-f) and *E*. *surinamensis* (g-j), demonstrating examples of the hybridization patterns of different microsatellite-motif probes. The sex chromosomes and the signals for each type of microsatellite motif are shown in the images. Note the intense signals along the chromosomal arms of both species, although band-like signals are also evident in distinct chromosomes and in distinct positions in *E*. *surinamensis* cells. One chromosome is missing in (b). Bar = 5 μm.

Labeling of the mono-, di-, tri and tetra-nucleotide arrays in *G*. *assimilis* cells revealed a relatively uniform dispersed distribution of each on all of the chromosomes (Fig 3A–3F). Only the (CGG)_10_ signal was less intense in the centromeric regions (Fig 3D). Although scattered signals for these arrays were generally observed in *E*. *surinamensis* cells, some of the signals formed band-like patterns in distinct chromosomes and in distinct positions, depending on the repeat mapped, with the exception of the (CAA)_10_ and (GATA)_8_ arrays, which showed only a scattered distribution (Fig 3G–3J, Table 1).

**Table 1:** Chromosomal positions of the band-like signals for microsatellite arrays in the cricket *Eneoptera surinamensis*.

Microsatellite motif	Pair 1	Pair 2	Pair 3	X_1_	X_2_	Y
(A)_30_	S: p	c	c			L: i,sd
(C)_30_	S: p			c	c	S: p,sd
(AG)_10_	S: p; L: p			c	c	
(CA)_15_	S: p			c	c	L: i,sd
(CG)_15_	c; L: 2i			c	c	c; L: 2p,i,sd; S: 2p,sd
(TA)_15_	c; L: i	c,d	c	c	c	L: p,i,sd; S: sd
(CAC)_10_					S: sd	L: 2p
(CAG)_10_	c	c	c	c	c	S: p; S: sd
(CGG)_10_	c			c	c	c; L: p
(TAC)_10_	c; L: 2i			c	c	c; L: 2p,sd; S: p,sd
(GAG)_10_	c; L: i	c	c,d	c	c	L: p,i,sd; S: sd
(GAA)_10_	c			c	c	S: p,sd
(TAA)_10_	c			c	c	
(GACA)_4_	c; L: p			c	c	c; L: 2p,sd; S: p,sd

L = long arm; S = short arm; c = centromeric; p = proximal; i = interstitial; sd = subdistal; d = distal.

**Fig 4:**
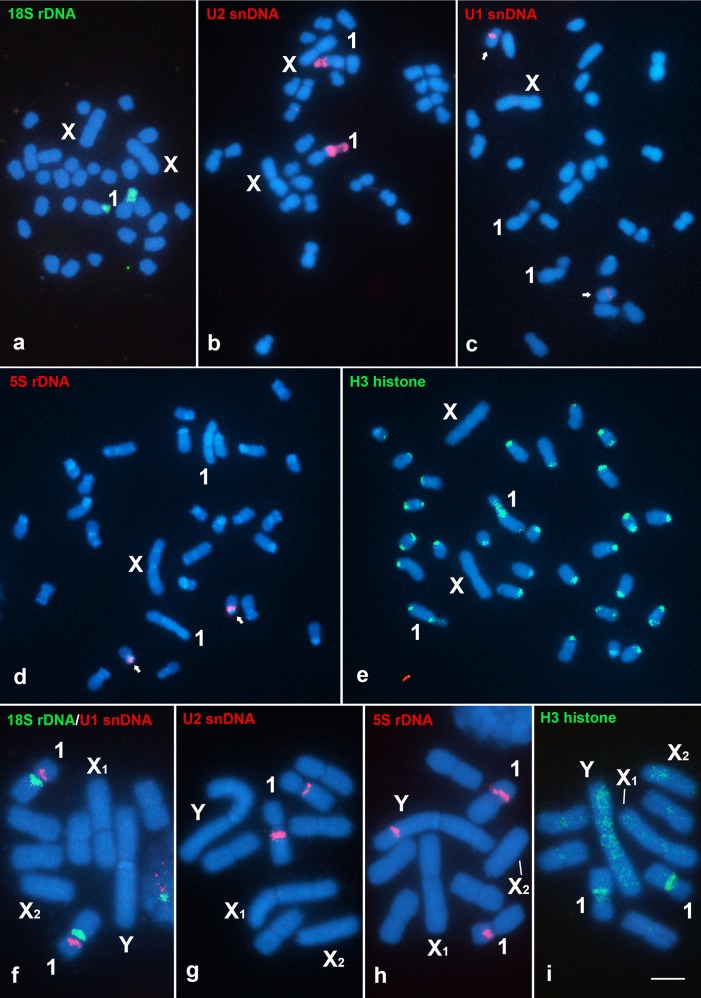
Chromosomal mapping of multigene families in mitotic cells of *G*. *assimilis* (a-e) and *E*. *surinamensis* (f-i). The sex chromosomes and the signals for each type of probe are shown in the images. Note the same chromosomal localization (pair 1) of 18S rDNA and U2 snDNA in both species and the signals for all of the multigene families in the pair 1 of *E*. *surinamensis*. Also, note the multiple and scattered signals of the H3 histone gene (e, i). The arrows in (c, d) indicate the chromosomes bearing signals. Bar = 5 μm.

**Fig 5:**
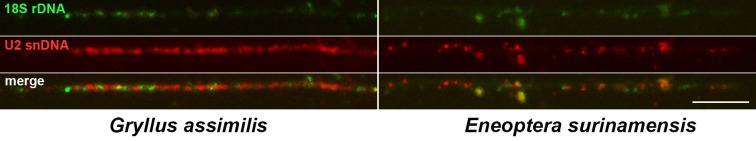
Fiber-FISH of the 18S rDNA and U2 snDNA probes in *G*. *assimilis* (a) and *E*. *surinamensis* (b). Note that these probes are co-localized and are interspersed in both species.

The FISH analyses revealed 18S rDNA and U2 snDNA signals on the pair 1 of *G*. *assimilis*, which were co-localized in the arm containing the secondary constriction (Fig 4A and 4B), whereas signals for 5S rDNA and U1 snDNA clusters were observed in the pericentromeric and proximal areas, respectively, of small subtelocentric chromosome pairs; however, due to slight differences in these chromosomes, it was not possible to precisely identify them (Fig 4C and 4D). The H3 histone signal occurred in evident bands in the terminal regions of some of the autosomes, whereas no H3 histone signal was observed on the X chromosome (Fig 4E). In *E*. *surinamensis* cells, labeling for all of the multigene families revealed clusters in the pair 1, and the 18S rDNA, U2 snDNA and H3 histone signals were coincident with the secondary constriction (Fig 4F, 4G and 4I), whereas the 5S rDNA and U1 snDNA signals were observed on the short arm (Fig 4F and 4H). However, an additional cluster of 5S rDNA labeling was observed in the interstitial region of the long arm of the neo-Y chromosome (Fig 4H), and a faint H3 histone signal was observed along all of the chromosomes, which was more evident in the neo-Y chromosome (Fig 4I). Fiber-FISH using probes for 18S rDNA and U2 snDNA, which were co-localized in the secondary constriction of the pair 1 chromosomes in metaphasic chromosomes in both species, revealed that these two sequences are interspersed in similar patterns (Fig 5).

The FCM analyses revealed that the mean genome size (1C) of *G*. *assimilis* cells is 2.13 pg (male: 2.06 pg and female: 2.21 pg), whereas *E*. *surinamensis* presents approximately 2.5 times greater DNA content, with a mean value of 5.54 pg (male: 5.44 pg and female: 5.65 pg) (see S1 Fig). Thus, the mean genome size of *G*. *assimilis* is 2.08 Gb and that of *E*. *surinamensis* is 5.42 Gb. The greater size of the genomes of the females of both species is certainly due to the presence of two copies of the X chromosome in female *G*. *assimilis* cells and the presence of two copies for the X_1_ and X_2_ chromosomes in female *E*. *surinamensis* cells.

## Discussion

### Karyotypes and general organization of repetitive DNA

The two cricket species examined in this study have highly divergent karyotypes. Considering that fusions are more frequent in Orthopteran chromosomal evolutionary history (for example, see [8,9,59]), leading to a reduced diploid number, the karyotype of *E*. *surinamensis* could be considered more evolved relative to that of *G*. *assimilis*. Repetitive DNAs can be relevant markers for tracing the evolution of the karyotypes and genomes of eukaryotes, but there is little information concerning the sizes of cricket genomes or the organization of their repetitive DNAs and their possible role in chromosomal evolution [13,17,38,39].

The diploid number and the sex system of *G*. *assimilis* observed in this study is identical to that previously described [40] and resembles that of most of the other *Gryllus* species that have been analyzed [10,13,60,61,62]. However, reduction of the diploid number has been observed in this genus [10], which includes the polymorphic condition of *G*. *assimilis* [11]. The main difference observed here in relation to other *Gryllus* karyotypes is the chromosomal morphology and the chromosome bearing the evident secondary constriction (see above references), suggesting that although *Gryllus* exhibits great karyotypic stability, small structural rearrangements may have occurred during the evolution of the genus. The diploid number observed in *E*. *surinamensis* (2n = 9) is well established in this species, having been observed in certain populations [63], and considering that a higher number of diploid chromosomes is more common in Gryllidae crickets [9,12,64], the karyotype of this species appears to be highly rearranged. Autosomal and autosomal/sex chromosome reshuffling most likely caused the significant reduction in the diploid number and the origin of a multiple sex system (neo-X_1_X_2_Y) with large chromosomes. It is difficult to specify the evolutionary causes or the order of the specific chromosomal rearrangements that led to the origin of the extreme karyotype of *E*. *surinamensis*, due to its extensive reorganization and the scarce phylogenetic and chromosomal information available for crickets. However, Robertsonian translocations (Rb-translocation), tandem fusions and pericentric inversions are most likely involved. In all cases of Rb-translocations or tandem fusions, the telomeres appeared to be either lost during the chromosomal rearrangement or eliminated during chromosome differentiation and no internal telomere sites are observed, as indicated by our FISH-based mapping of the telomere motif, which revealed signals only in the actual telomeres. A similar condition was reported in a small number of studied grasshoppers with derived karyotypes in which centric fusion or Rb-translocation occurred [15,16]. However, due to the limited sensitivity of the classical FISH technique, the possibility of small internal telomeric sites cannot be completely ruled out. In *Gryllus*, if the karyotype experienced inversions, as justified by distinct chromosomal morphologies relative to those of other species, these rearrangements occurred without the involvement of telomeres or the telomeres were lost. An alternative explanation for this condition is that *G*. *assimilis* has a non-rearranged karyotype compared with those of other species of the genus, which should be evaluated using a better species sampling.

There is little information concerning heterochromatin distribution in crickets, but in the genus *Gryllus*, the occurrence of mainly centromeric and terminal heterochromatic blocks has been observed in certain species, i.e., *G*. *bimaculatus*, *G*. *argentinus* and *Gryllus* sp. [13,60,62], as observed in this study in *G*. *assimilis*. In contrast, in *E*. *surinamensis*, the C-positive blocks were most organized in the centromeric region of the autosomes, as well as in other chromosomal regions, with extensive spreading in the neo-Y chromosome. These patterns, including those observed in *Cycloptiloides americanus* [17], suggest the intense reorganization of the C-positive blocks in the rearranged karyotypes. These data indicate that the heterochromatin chromosomal dynamic is more intense in crickets than in other Orthoptera, such as grasshoppers, in which pericentromeric C-positive blocks are most commonly observed [65,66], although other species should be studied. The data obtained through fluorochrome staining suggests the occurrence of repetitive DNA families with distinct richness in A+T or G+C base pairs, which in *E*. *surinamensis* are G+C-rich, whereas in *G*. *assimilis*, they are mainly neutral for A+T or G+C, suggesting the restructuring of repetitive DNA families in their two karyotypes. This data was confirmed in *E*. *surinamensis* through genomic analysis that revealed the occurrence of mainly G+C-rich satDNAs (Palacios-Gimenez et al., unpublished data). In another *Gyllus* species, i.e., *G*. *bimaculatus*, some terminal heterochromatic blocks were enriched for two A+T-rich satDNAs families, one of which is also present in other *Gryllus* species, including *G*. *rubens* and *Gryllus* sp [13]. The occurrence of A+T-rich repetitive families in the genome of *G*. *assimilis* could not be ruled out, and DAPI^+^ blocks could not be observed in this organism due to the limits of resolution.

The chromosomal organization of repetitive DNAs obtained through C-banding was corroborated by *C*_*0*_ *t*-DNA mapping that revealed a similar pattern, as well as additional regions enriched in repetitive sequences, such as the centromere of the neo-X_1_ chromosome of *E*. *surinamensis* and some faintly labelled dispersed areas. In other Orthopteran species, the *C*_*0*_ *t*-DNA fraction is also enriched in C-positive autosomal blocks, but divergent patterns have been observed in the sex chromosomes, including the derivate neo-sex chromosomes, as observed in *E*. *surinamensis*. For example, enrichment of repetitive DNAs in the neo-Y element of the grasshopper *Ronderosia bergi* [16] and the X_2_ element of the cricket *C*. *americanus* [17] have been reported, suggesting a role of this genomic fraction in sex chromosome differentiation, causing chromatin to change into heterochromatin. The accumulation of repetitive DNAs is a common feature during the differentiation of the Y or W sex chromosomes, and it has been documented in various animal and plant species, such as *Rumex* species [27,67], *Silene latifolia* [30], lizard species [68], *Schistosoma mansoni* [69] and *Drosophila miranda* [70,71]. However, in some grasshopper species, the *C*_*0*_ *t*-DNA fraction is restricted to the centromeres of the sex chromosomes [15].

The FISH mapping results regarding the microsatellites suggest that a major strong differentiation in the chromosomal organization of the karyotypes of the two species occurred. Although the repeats are mainly dispersed in both euchromatin and heterochromatin, clustering was observed, with the repeats forming a band-like pattern, particularly those in the heterochromatic regions of the pair 1 and sex chromosomes of *E*. *surinamensis*. This result suggests that the reduction in the diploid number was followed by the “compartmentalization” of the microsatellites in the genome, with these sequences being involved in the heterochromatin, including those of the sex chromosomes. The accumulation of some of the microsatellite arrays in the neo-Y chromosomes of *E*. *surinamensis* was expected, considering that they are non-recombining elements, as suggested by the lack of contact of these three chromosomes during meiosis [8], and this data is consistent with that observed in other species [72,73,74,75]. As previously observed in the grasshopper *R*. *bergi*, the microsatellites of *E*. *surinamensis* are clearly involved in the differentiation between sex chromosomes [16]. Although poorly studied in Orthoptera, the dispersal or specific chromosomal distribution of the microsatellite arrays in band-like patterns was recently reported in other species, such as the cricket *C*. *americanus* [17] and the grasshoppers *Abracris flavolineata* [76], *R*. *bergi* [16], *Locusta migratoria* and *Eyprepocnemis plorans* [77]. However, next generation sequencing analysis revealed uneven and nonrandom localization of microsatellites in *L*. *migratoria* and *E*. *plorans*, with the dinucleotide motifs predominantly associated with other repetitive DNAs, such as the histone gene spacer, rDNA intergenic spacers (IGSs) and transposable elements [77]. A similar pattern was observed in *E*. *surinamensis*, in which some of the microsatellite arrays that are distributed in a band-like pattern are co-localized with some of the multigene families and the anonymous *C*_*0*_ *t*-DNA fraction.

The increased size of the genome of *E*. *surinamensis* compared with that of *G*. *assimilis* is notable, and considering the little information available concerning the size of the genomes of crickets [38,39], this is apparently a derivate characteristic. The increased size of this genome could be attributed to the amplification, spreading and accumulation of repetitive DNAs in specific chromosomal regions, such as observed for *C*_*0*_ *t-*DNA and some satellite repeats (Palacios-Gimenez et al., unpublished data), besides some microsatellites. Although, there is no evidence that the reduction in the diploid number contributed to this process. This process is more evident in the derivate sex chromosomes, most likely due to the non-recombinant state of the neo-Y chromosome and the restricted recombination of the neo-X_1_ and neo-X_2_ chromosomes in the female germline. Additionally, the structural mutation rates of the neo-Y chromosomes of some species are more rapid than those of other genomic regions, suggesting the reduced efficacy of natural selection in this chromosome [18,78,79,80], which is likely to occur in the genome of *E*. *surinamensis*.

### The multigene families

No variation in the location and number of clusters of 18S rDNA and U2 snDNA was observed, both of which are conserved on the pair 1. In grasshoppers [81,82] and other insects, such as Lepidoptera [83], Coleoptera [84] and Heteroptera [85], extremely variable patterns of the major rDNA was observed contrasting with this study. Remarkably, the pattern of U2 snDNA on the pair 1 is highly conserved in some grasshopper species [15,48] and in the cricket *C*. *americanus* (Mogoplistidae) [17]. Conservation of the number of U2 snDNA clusters is observed also in other groups, such as *Gymnotus* species [86], in contrast to the case in some of the other fish species of the Batrachoididae family, in which the U2 snDNA signals appear to be widely scattered [87]. The 18S rDNA and U2 snDNA signals were located in the same sites and exhibited the same association in the two species, which was supported by the fiber-FISH results, which may indicate an ancestral characteristic in Gryllidae that was maintained despite the exceptional chromosomal divergence. However, other species in the family should be studied to confirm this hypothesis. The association or interspersion of distinct multigene families has been reported in distinct groups, but the significance of these patterns is not clear and they have no apparent selective advantage [45,82,84,88,89,90,91].

The presence of 5S rDNA and U1 snDNA in the small chromosomes of *G*. *assimilis* in contrast to their presence in the pair 1 of *E*. *surinamensis* may be the consequence of large-scale chromosomal rearrangements, as mentioned above, which translocated these genes to the same chromosome. However, the localization of 5S rDNA in the neo-Y chromosome of *E*. *surinamensis* suggests both amplification and transposition or even further chromosomal rearrangement involving sex chromosomes and ancestral autosomes bearing this gene, as has been suggested for other Orthopteran species that have neo-sex chromosomes [15,16,17]. The presence of this gene in the neo-Y chromosomes and absence in the neo-X chromosomes reinforce the differentiation between the neo-sex chromosomes of *E*. *surinamensis* that was established after the chromosomal rearrangements occurred.

Contrary to what was observed for rDNAs and U snDNAs, significant dispersion of the H3 histone gene occurred in *G*. *assimilis* and *E*. *surinamensis*, with differentially spread patterns observed in the two species, with these DNA elements clearly in blocks in *G*. *assimilis* and faint signals observed in *E*. *surinamensis*. These results suggest the independent amplification/dispersion and dynamism of the H3 histone genes at the chromosomal level. Multiple H3 histone sites, in blocks such as observed in *G*. *assimilis*, have rarely been reported in grasshoppers, such as *Abracris flavolineata* [48], *Dichromatos lilloanus* [15] and *Ronderosia bergi* [16]. Although, this pattern is less common, being commonly observed an interstitial cluster in the pair 8 a highly conserved character [92]. Our data suggest that the organization and evolution of the H3 histone genes may be more dynamic than was previously reported and that this dispersal in *G*. *assimilis* and *E*. *surinamensis* could be due to other mechanisms, such as association with other repetitive DNAs (transposons or satDNAs), ectopic recombination or extrachromosomal circular DNA (eccDNA), as has been proposed for rDNAs [19,81,93]. An alternative hypothesis for the dispersal of H3 histone genes is the similarity of the sequences of these genes with those of unknown repetitive DNAs. Remarkably, a main cluster of H3 histone genes occurs in the pair 1, as is the case for the other four multigene families investigated in this study. This arrangement of certain multigene families in one unique bivalent chromosome has not yet been described and could be result of the large-scale rearrangements observed in the *E*. *surinamensis* karyotype.

In conclusion, our study provided an opportunity to explore the evolutionary dynamics of repetitive DNAs in two non-model species and its possible involvement in karyotypic organization and genome increasing. As we suggested some of the differences between the two evaluated karyotypes were most likely a consequence of the evolution and distribution of repetitive DNA that were accumulated in specific chromosomal regions. Whereas certain characteristics that these species shared could indicate their ancestral relationship. Moreover, the distinct patterns of the classes of repetitive sequences that were mapped in this study suggests that the differential amplification and accumulation of this class of DNA occurred mainly in the neo-Y chromosomes, highlighting the differentiation of the neo-sex chromosomes of *E*. *surinamensis*.

## Supporting Information

S1 Fig (a,b) Examples of histograms showing the relative nuclear DNA content from cerebral ganglion cells stained with PI for *S*. *xantotricha* (internal standard), *G*. *assimilis* and *E*. *surinamensis*. (c) Genome size estimation in three replicates form each sex for species studied in this paper and for the female of *S*. *xantotricha* used as internal standard. The values are in pictogram (pg). (PDF)

## Abbreviations

2n: diploid number
*C*_*0*_*t*-DNA: *C*_*0*_ is the initial concentration of single-stranded DNA in mol/l and t is the reannealing time in seconds
CMA_3_: Chromomycin A_3_
DA: Distamicyn
DAPI: 4’, 6-Diamidino-2-phenylindole
FCM: Flow cytometry
FISH: Fluorescence in situ hybridization
PCR: Polymerase chain reaction
PI: Propidium iodide
Rb-translocation: Robertsonian translocation
rDNA: Ribosomal DNA
rRNA: Ribosomal RNA
snRNA: Small nuclear RNA

## References

[1] Grimaldi D, Engel MS. Evolution of the insects Cambridge University Press, New York; 2005.

[2] Eades DC, Otte D, Cigliano MM, Braun H. Orthoptera species file. Version 5.0/5.0. [21/6/2015]. Availible: http://Orthoptera.speciesfile.org.

[3] Song H, Amédégnato C, Cigliano MM, Desutter-Grandcolas L, Heads SW, Huang Y, et al 300 million years of diversification: elucidating the patterns of orthopteran evolution based on comprehensive taxon and gene sampling. Cladistics. 2015 2 4 10.1111/cla.12116 34753270

[4] Warchalowska-Śliwa E. Karyotype characteristics of katydid Orthopterans (Ensifera, Tettigoniidae) and remarks on their evolution at different taxonomic levels. Folia Biol. 1998;4: 143–176.

[5] Ferreira A, Mesa A. Cytogenetics studies in Brazilian species of Pseudophyllinae (Orthoptera: Tettigoniidae): 2n(♂) = 35 and FN = 35 the probable basic and ancestral karyotype of the family Tettigoniidae. Neotr Entomol. 2010;39: 590–594. 10.1590/s1519-566x2010000400019 20877996

[6] Grzywacz B, Heller KG, Lehmann AW, Warchałowska-Śliwa E, Lehmann GUC. Chromosomal diversification in the flightless Western Mediterranean bush cricket genus Odontura (Orthoptera: Tettigoniidae: Phaneropterinae) inferred from molecular data. J Zoolog Syst Evol Res. 2014;52: 109–118.

[7] Sáez FA. An extreme karyotype in an orthopteran insect. Amer Nat. 1957;91: 259–264.

[8] White MD. Animal cytology and evolution 3re ed. Cambridge Univ. Press, London; 1973.

[9] Hewitt GM. Grasshoppers and crickets Animal cytogenetics. vol 3: Insecta 1. Orthoptera.Gebrüder Borntraeger, Berlin; 1979.

[10] Handa SM, Mittal OP, Sehgal S. Cytology of ten species of crickets from Chandigarh (lndia). Cytologia. 1985;50: 711–72.

[11] Zefa E. Autosomal rearrangement in *Gryllus assimilis* Fabricius, 1775 (Orthoptera, Gryllidae). Genet Mol Biol. 1999;22: 333–336.

[12] Ferreira A, Cella DM. Chromosome structure of *Eneoptera surinamensis* (Orthoptera, Grilloidea, Eneopterinae) as revealed by C, NOR and N-banding techniques. Chromosome Sci. 2006;9: 47–51.

[13] Yoshimura A, Nakata A, Mito T, Noji S. The characteristics of karyotype and telomeric satellite DNA sequences in the cricket, *Gryllus bimaculatus* (Orthoptera, Gryllidae). Cytogenet. Genome Res. 2006;112: 329–336. 16484791 10.1159/000089889

[14] Castillo ER, Martí DA, Bidau CJ. Sex and neo-sex chromosomes in Orthoptera: a review. J Orthopt Res. 2010;19: 213–231.

[15] Palacios-Gimenez OM, Castillo ER, Martí DA, Cabral-de-Mello DC. Tracking the evolution of sex chromosome systems in Melanoplinae grasshoppers through chromosomal mapping of repetitive DNA sequences. BMC Evol Biol. 2013;13: 167 10.1186/1471-2148-13-167 23937327 PMC3751140

[16] Palacios-Gimenez OM, Marti DA, Cabral-de-Mello DC. Neo-sex chromosomes of *Ronderosia bergi*: insight into the evolution of sex chromosomes in grasshoppers. Chromosoma. 2015 1 21 10.1007/s00412-015-0505-1 25605041

[17] Palacios-Gimenez OM, Cabral-de-Mello DC. Repetitive DNA chromosomal organization in the cricket *Cycloptiloides americanus*: a case of the unusual X_1_X_2_0 sex chromosome system in Orthoptera. Mol Genet Genomics. 2015;290: 623–631. 10.1007/s00438-014-0947-9 25373534

[18] Charlesworth B, Sniegowski P, Stephan W. The evolutionary dynamics of repetitive DNA in eukaryotes. Nature. 1994;371: 215–220. 8078581 10.1038/371215a0

[19] Nei M, Rooney AP. Concerted and birth-and-death evolution of multigene families. Annu Rev Genet. 2005;39: 121–52. 16285855 10.1146/annurev.genet.39.073003.112240 PMC1464479

[20] Biémont C, Vieira C. Genetics: junk DNA as an evolutionary force. Nature. 2006;443: 521–524. 17024082 10.1038/443521a

[21] Richard GF, Kerrest A, Dujon B. Comparative genomics and molecular dynamics of DNA repeats in eukaryotes. Microbiol Mol Biol Rev. 2008;72: 686–727. 10.1128/MMBR.00011-08 19052325 PMC2593564

[22] López-Flores I, Garrido-Ramos MA. The repetitive DNA content of eukaryotic genomes In: Garrido-Ramos MA editor. Repetitive DNA. Genome Dyn. Basel, Karger, vol 7; 2012 pp. 1–28. 10.1159/000337118 22759811

[23] Doolittle WF, Sapienza C. Selfish genes, the phenotype paradigm and genome evolution. Nature. 190;284: 601–603. 6245369 10.1038/284601a0

[24] Orgel LE, Crick FHC. Selfish DNA: the ultimate parasite. Nature. 1980;284: 604–607. 7366731 10.1038/284604a0

[25] Charlesworth B. The evolution of chromosomal sex determination and dosage compensation. Curr Biol. 1996;6: 149–162. 8673462 10.1016/s0960-9822(02)00448-7

[26] Skaletsky H, Kuroda-Kawaguchi T, Minx PJ, Cordum HS, Hillier L, Brown LG, et al The male-specific region of the human Y chromosome is a mosaic of discrete sequence classes. Nature. 2003;423: 825–837. 12815422 10.1038/nature01722

[27] Navajas-Pérez R, de la Herrán R, Jamilena M, Lozano R, Ruiz Rejón C, Ruiz Rejón M, Garrido-Ramos MA (2005). Reduced rates of sequence evolution of Y-linked satellite DNA in *Rumex* (Polygonaceae). J Mol Evol. 2005;60: 391–399. 15871049 10.1007/s00239-004-0199-0

[28] Eickbush TH, Eickbush DG. Finely orchestrated movements: evolution of the ribosomal RNA genes. Genetics. 2007;175: 477–485. 17322354 10.1534/genetics.107.071399 PMC1800602

[29] Matsunaga S. Junk DNA promotes sex chromosome evolution. Heredity. 2009;102: 525–526. 10.1038/hdy.2009.36 19337304

[30] Kejnovsky E, Hobza R, Cermak T, Kubat Z, Vyskot B. The role of repetitive DNA in structure and evolution of sex chromosomes in plants. Heredity. 2009;102: 533–541. 10.1038/hdy.2009.17 19277056

[31] Kidwell MG, Lisch D. Transposable elements as sources of variation in animals and plants. Proc Natl Acad Sci USA. 1997;94: 7704–7711. 9223252 10.1073/pnas.94.15.7704 PMC33680

[32] Gregory TR. The evolution of the genome Elsevier Academic Press, San Diego CA USA; 2005.

[33] Lynch M. The origins of genome architecture Sinauer Associates, Inc. Publishers, Sunderland, MA, USA; 2007.

[34] Rasch EM, Rasch RW. Cytophotometric determination of genome size for two species of cave crickets (Orthoptera, Rhaphidophoridae). J Histochem Cytochem. 1991;29: 885.

[35] Westerman M, Barton NH, Hewitt GM. Differences in DNA content between two chromosomal races of the grasshopper *Podisma pedestris* . Heredity. 1987;58: 221–228.

[36] Wang X, Fang X, Yang P, Jiang X, Jiang F, Zhao D, et al The locust genome provides insight into swarm formation and long-distance flight. Nature Commun. 2014;5: 2957. 24423660 10.1038/ncomms3957 PMC3896762

[37] Camacho JPM, Ruiz-Ruano FJ, Martín-Blázquez R, López-León MD, Cabrero J, Lorite P, et al A step to the gigantic genome of the desert locust: chromosome sizes and repeated DNAs. Chromosoma. 2015;124: 263–275. 10.1007/s00412-014-0499-0 25472934

[38] Hanrahan SJ, Johnston S. New genome size estimates of 134 species of arthropods. Chromosome Res. 2011;19: 809–823. 10.1007/s10577-011-9231-6 21877225

[39] Gregory TR. Animal Genome Size Database. 2015. Available: http://www.genomesize.com.

[40] Baumgartner WJ. Some evidence for the individuality of the chromosomes. Biol Bull. 1904;8: 1–23.

[41] Webb GC, White MJD, Contreras N, Cheney J. Cytogenetics of the parthogenetic grasshopper *Warramaba* (formerly *Moraba*) *virgo* and its bisexual relatives. IV. Chromosome banding studies. Chromosoma. 1978;67: 309–339.

[42] Sumner AT. A simple technique for demonstrating centromeric heterochromatin. Exp Cell Res. 1972;75: 304–306. 4117921 10.1016/0014-4827(72)90558-7

[43] Schweizer D, Mendelak M, White MJD, Contreras N. Cytogenetics of the parthenogenetic grasshopper *Warramaba virgo* and its bisexual relatives. X. Pattern of fluorescent banding. Chromosoma. 1983;88: 227–236.

[44] Sambrook J, Russel DW. Molecular cloning: A laboratory manual Cold Spring Harbor, NY: Cold Spring Harbor Laboratory Press; 2001.

[45] Cabral-de-Mello DC, Moura RC, Martins C. Chromosomal mapping of repetitive DNAs in the beetle *Dichotomius geminatus* provides the first evidence for an association of 5S rRNA and histone H3 genes in insects, and repetitive DNA similarity between the B chromosome and A complement. Heredity. 2010;104: 393–400. 10.1038/hdy.2009.126 19756039

[46] Colgan DJ, McLauchlan A, Wilson GDF, Livingston SP, Edgecombe GD, Macaranas J, et al Histone H3 and U2 snRNA DNA sequences and arthropod molecular evolution. Austral J Zool. 1998;46: 419–437.

[47] Cabral-de-Mello DC, Valente GT, Nakajima RT, Martins C. Genomic organization and comparative chromosome mapping of the U1 snRNA gene in cichlid fish, with an emphasis in *Oreochromis niloticus* . Chromosome Res. 2012;20: 279–292. 10.1007/s10577-011-9271-y 22234547

[48] Bueno D, Palacios-Gimenez OM, Cabral-de-Mello DC. Chromosomal mapping of repetitive DNAs in *Abracris flavolineata* reveal possible ancestry for the B chromosome and surprisingly H3 histone spreading. PLoS ONE. 2103;8: e66532. 10.1371/journal.pone.0066532 PMC3694960 23826099

[49] Zwick MS, Hanson RE, McKnight TD, Nurul-Islam-Faridi M, Stelly DM. A rapid procedure for the isolation of *C* _*0*_ *t*–1 DNA from plants. Genome. 1997;40: 138–142. 18464813 10.1139/g97-020

[50] Ijdo JW, Wells RA, Baldini A, Reeders ST. Improved telomere detection using a telomere repeat probe (TTAGGG)n generated by PCR. Nucleic Acids Res. 1991;19: 4780 1891373 10.1093/nar/19.17.4780 PMC328734

[51] Pinkel D, Straume T, Gray JW. Cytogenetic analysis using quantitative, high sensitivity, fluorescence hybridization. Proc Natl Acad Sci USA. 1986;83: 2934–2938. 3458254 10.1073/pnas.83.9.2934 PMC323421

[52] de Barros AV, Sczepanski TS, Cabrero J, Camacho JPM, Vicari MR, Artoni RF. Fiber FISH reveals different patterns of high-resolution physical mapping for repetitive DNA in fish. Aquaculture. 2011;322: 47–50.

[53] Camacho JPM, Cabrero J, López-León MA, Cabral-de-Mello DC, Ruiz-Ruano FJ. Grasshoppers (Orthoptera) In: Sharakhov V editor. Protocols for cytogenetic mapping of arthropod genomes. Boca Ratton, FL, USA: CRC Press; 2015 pp. 381–438.

[54] Lopes DM, Carvalho CR, Clarindo WR, Praça MM, Tavares MG. Genome size estimation of three stingless bee species (Hymenoptera, Meliponinae) by flow cytometry. Apidologie. 2009;40: 517–523.

[55] Otto F. DAPI staining of fixed cells for high-resolution flow cytometry of nuclear DNA. In: Darzynkiewicz Z, Crissman HA editors. Methods in cell biology. Vol 33; 1990. pp. 105–110. 10.1016/s0091-679x(08)60516-6 1707478

[56] Loureiro J, Rodriguez E, Doležel J, Santos C. Comparison of four nuclear isolation buffers for plant DNA flow cytometry. Ann Bot. 2006;98: 679–689. 16820407 10.1093/aob/mcl141 PMC2803574

[57] Loureiro J, Rodriguez E, Doležel J, Santos C. Flow cytometric and microscopic analysis of the effect of tannic acid on plant nuclei and estimation of DNA content. Ann Bot. 2006;98: 515–527. 16820406 10.1093/aob/mcl140 PMC2803573

[58] Shapiro HM. Practical flow cytometry, 4th ed., John Wiley & Sons, New Jersey; 2003.

[59] Mesa A, Ferreira A, Carbonell CS. Cariologia de los acridios neotropicales: estado actual de su conocimiento y nuevas contribuciones. Ann Soc Entomol Fr (NS). 1982; 18: 507–526.

[60] Drets ME, Stoll M. C-banding and non-homologous associations in *Gryllus argentinus* . Chromosoma. 1974;48: 367–390. 4448110 10.1007/BF00290994

[61] Lamborot M, Alvarez-Sarret E. The chromosomes of *Gryllus* field cricket populations in central Chile (Insecta: Grylloptera: Gryllidae).Can J Zoolog. 1985;63: 2626–2631.

[62] Yoshimura A. Karyotypes of two American field crickets, *Gryllus rubens* and *Gryllus* sp. (Orthoptera: Gryllidae). Entomol Sci. 2005;8: 219–222.

[63] Zefa E, Cordeiro J, Blauth M, Piumbini M, Silva AF, Costa MKM, Martins LP. Expanding the geographic cytogenetic studies in the bush crickets *Eneoptera surinamensis* (De Geer, 1773) (Orthoptera, Gryllidae, Eneopterinae) from Brazilian Atlantic and Amazon Forest. Zootaxa. 2014;3860: 396–400. 10.11646/zootaxa.3860.4.7 25283215

[64] Mesa A, Bran EJ. Acerca de los cromosomas de *Eneoptera surinamensis* . An II Cong Latino-Americ Zol S Paulo. 1964;I: 9–16.

[65] Santos JL, Arana P, Giráldez L. Chromosome C-banding patterns in Spanish Acridoidea. Genetica. 1983;61: 65–74.

[66] John B, King M. The inter-relationship between heterochromatin distribution and chiasma distribution. Genetica. 1985; 66: 183–194.

[67] Steflova P, Tokan V, Vogel I, Lexa M, Macas J, Novak P, et al Contrasting patterns of transposable element and satellite distribution on sex chromosomes (XY_1_Y_2_) in the dioecious plant *Rumex acetosa* . Genome Biol Evol. 2013; 5: 769–782. 10.1093/gbe/evt049 23542206 PMC3641822

[68] Pokorná M, Kratochvíl L, Kejnovský E. Microsatellite distribution on sex chromosomes at different stages of heteromorphism and heterochromatinization in two lizard species (Squamata: Eublepharidae: *Coleonyx elegans* and Lacertidae: *Eremias velox*). BMC Genet. 2011;12: 90 10.1186/1471-2156-12-90 22013909 PMC3215666

[69] Lepesant JMJ, Cosseau C, Boissier J, Freitag M, Portela J, Climent D, et al Chromatin structural changes around satellite repeats on the female sex chromosome in *Schistosoma mansoni* and their possible role in sex chromosome emergence. Genome Biol. 2012;13: R14 10.1186/gb-2012-13-2-r14 22377319 PMC3701142

[70] Steinemann S, Steinemann M. The enigma of Y chromosome degeneration: TRAM,a novel retrotransposonis preferentially located on the Neo-Y chromosome of *Drosophila miranda* . Genetics. 1997;145: 261–266. 9071582 10.1093/genetics/145.2.261 PMC1207793

[71] Steinemann S, Steinemann M. Retroelements: tools for sex chromosome evolution. Cytogenet Genome Res. 2005;110: 134–143. 16093665 10.1159/000084945

[72] Kubat Z, Hobza R, Vyskot B, Kejnovsky E. Microsatellite accumulation on the Y chromosome in *Silene latifolia* . Genome. 2008;51: 350–356. 10.1139/G08-024 18438438

[73] Kejnovský E, Michalovova M, Steflova P, Kejnovska I, Manzano S, Hobza R, et al Expansion of microsatellites on evolutionary young Y chromosome. PLoS One. 2013;8: e45519 10.1371/journal.pone.0045519 23341866 PMC3547029

[74] Matsubara K, Knopp T, Sarre SD, Georges A, Ezaz T. Karyotypic analysis and FISH mapping of microsatellite motifs reveal highly differentiated XX/XY sex chromosomes in the pink-tailed worm-lizard (*Aprasia parapulchella*, Pygopodidae, Squamata). Mol Cytogenet. 2013;6: 60 10.1186/1755-8166-6-60 24344753 PMC3905675

[75] Matsubara K, O'Meally D, Azad B, Georges A, Sarre SD, Graves JA, et al Amplification of microsatellite repeat motifs is associated with the evolutionary differentiation and heterochromatinization of sex chromosomes in Sauropsida. Chromosoma. 2015 7 21 10.1007/s00412-015-0531-z 26194100

[76] Milani D, Cabral-de-Mello DC. Microsatellite organization in the grasshopper *Abracris flavolineata* (Orthoptera: Acrididae) revealed by FISH mapping: remarkable spreading in the A and B chromosomes. PLoS ONE. 2014;9: e97956 10.1371/journal.pone.0097956 24871300 PMC4037182

[77] Ruiz-Ruano FJ, Cuadrado Á, Montiel EE, Camacho JPM, López-León MD. Next generation sequencing and FISH reveal uneven and nonrandom microsatellite distribution in two grasshopper genomes. Chromosoma. 2015;124: 221–234. 10.1007/s00412-014-0492-7 25387401

[78] Clark AG. The vital Y chromosome. Nature. 2014;508: 463–465. 10.1038/508463a 24759407

[79] Toups M, Veltsos P, Pannell JR. Plant sex chromosomes: lost genes with little compensation. Curr Biol. 2015;25: 427–429. 10.1016/j.cub.2015.03.054 25989086

[80] Bergero R, Qiu S, Charlesworth D. Gene loss from a plant sex chromosome system. Curr Biol. 2015;25: 1234–1240. 10.1016/j.cub.2015.03.015 25913399

[81] Cabrero J, Camacho JP. Location and expression of ribosomal RNA genes in grasshoppers: abundance of silent and cryptic loci. Chromosome Res. 2008;16: 595–607. 10.1007/s10577-008-1214-x 18431681

[82] Cabral-de-Mello DC, Cabrero J, López-León MD, Camacho JPM. Evolutionary dynamics of 5S rDNA location in acridid grasshoppers and its relationship with H3 histone gene and 45S rDNA location. Genetica. 2011;139: 921–931. 10.1007/s10709-011-9596-7 21755328

[83] Nguyen P, Sahara K, Yoshido A, Marec F. Evolutionary dynamics of rDNA clusters on chromosomes of moths and butterflies (Lepidoptera). Genetica. 2010;138: 343–354. 10.1007/s10709-009-9424-5 19921441

[84] Cabral-de-Mello DC, Oliveira SG, Moura RC, Martins C. Chromosomal organization of the 18S and 5S rRNAs and histone H3 genes in Scarabaeinae coleopterans: insights into the evolutionary dynamics of multigene families and heterochromatin. BMC Genet. 2011;12: 88 10.1186/1471-2156-12-88 21999519 PMC3209441

[85] Panzera Y, Pita S, Ferreiro MJ, Ferrandis I, Lages C, Pérez R, et al High dynamics of rDNA cluster location in kissing bug holocentric chromosomes (Triatominae, Heteroptera). Cytogenet Genome Res. 2012;138: 56–67. 10.1159/000341888 22907389

[86] Utsunomia R, Scacchetti PC, Pansonato-Alves JC, Oliveira C, Foresti F. Comparative chromosome mapping of U2 snRNA and 5S rRNA genes in *Gymnotus* species (Gymnotiformes, Gymnotidae): evolutionary dynamics and sex chromosome linkage in *G*. *pantanal* . Cytogenet Genome Res. 2014;142: 286–292. 10.1159/000362258 24776647

[87] Úbeda-Manzanaro M, Merlo MA, Palazón JL, Cross I, Sarasquete C, Rebordinos L. Chromosomal mapping of the major and minor ribosomal genes, (GATA)n and U2 snRNA gene by double-colour FISH in species of the Batrachoididae family. Genetica. 2010;138: 787–794. 10.1007/s10709-010-9460-1 20440541

[88] Pelliccia F, Barzotti R, Bucciarelli E, Rocchi A. 5S ribosomal and U1 small nuclear RNA genes: a new linkage type in the genome of a crustacean that has three different tandemly repeated units containing 5S ribosomal DNA sequences. Genome. 2001;44: 331–335. 11444690 10.1139/gen-44-3-331

[89] Manchado M, Zuasti E, Cross I, Merlo A, Infante C, Rebordinos L. Molecular characterization and chromosomal mapping of the 5S rRNA gene in *Solea senegalensis*: a new linkage to the U1, U2, and U5 small nuclear RNA genes. Genome. 2006;49: 79–86. 16462904 10.1139/g05-068

[90] Vierna J, Jensen KT, Martínez-Lage A, González-Tizón AM. The linked units of 5S rDNA and U1 snDNA of razor shells (Mollusca: Bivalvia: Pharidae). Heredity. 2011;107: 127–142. 10.1038/hdy.2010.174 21364693 PMC3178405

[91] Anjos A, Ruiz-Ruano FJ, Camacho JPM, Loreto V, Cabrero J, de Souza MJ, Cabral-de-Mello DC. U1 snDNA clusters in grasshoppers: chromosomal dynamics and genomic organization. Heredity. 2015;114: 207–219. 10.1038/hdy.2014.87 25248465 PMC4815631

[92] Cabrero J, López-León MD, Teruel M, Camacho JP. Chromosome mapping of H3 and H4 histone gene clusters in 35 species of acridid grasshoppers. Chromosome Res. 2009;17: 397–404. 10.1007/s10577-009-9030-5 19337846

[93] Cohen S, Agmon N, Sobol O, Segal D. Extrachromosomal circles of satellite repeats and 5S ribosomal DNA in human cells. Mobile DNA. 2010;1: 11 10.1186/1759-8753-1-11 20226008 PMC3225859

