# Supplementary material for: Contrasting the Chromosomal Organization of Repetitive DNAs in Two Gryllidae Crickets with Highly Divergent Karyotypes
Source: PLoS One. 2015 Dec 2;10(12):e0143540. doi: 10.1371/journal.pone.0143540 (PMC4667936; doi:10.1371/journal.pone.0143540)
Supplement: S1 Fig — (PDF) [file pone.0143540.s001.pdf]

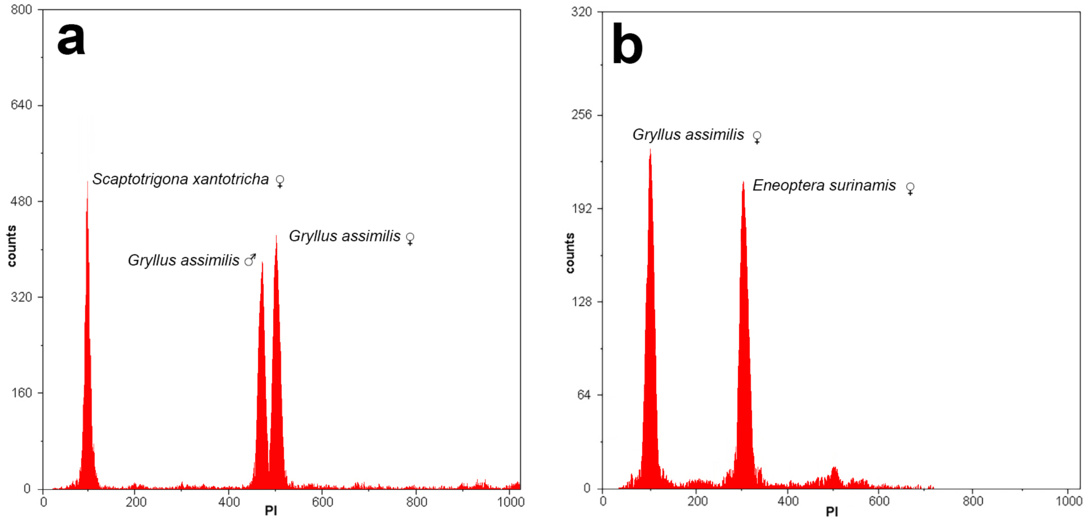

c

Sample	Replicate 1	Replicate 2	Replicate 3	Mean of DNA content (1C)
Scaptotrigona xantotricha (female)	0.44	0.44	0.44	0.44 pg
Gryllus assimilis (male)	2.07	2.075	2.05	2.06 pg
G. assimilis (female)	2.20	2.22	2.21	2.21 pg
Eneoptera surinamensis (male)	5.47	5.45	5.41	5.44 pg
E. surinamensis (female)	5.6	5.7	5.65	5.65 pg
